# The effect of external marking on the behaviour of the common pill woodlouse *Armadillidium vulgare*

**DOI:** 10.3897/zookeys.176.2375

**Published:** 2012-03-20

**Authors:** Táňa Drahokoupilová, Ivan Hadrián Tuf

**Affiliations:** 1 Department of Ecology and Environmental Sciences, Faculty of Science, Palacky University, Svobody 26, CZ-77200, Olomouc, Czech Republic

**Keywords:** Diurnal activity, external marking, influence on behaviour, daily pattern, Isopoda, Oniscidea

## Abstract

Zoologists distinguish individual animals using marking techniques. Generally they test the potential influence of marking on survival only; the influence on behaviour is usually neglected. We evaluated the influence of two external marking techniques (nail polish and queen-bee marker) on the behaviour of common pill woodlouse, *Armadillidium vulgare*. The behaviour was examined from two points of view: (1) activity during 24 hours and (2) specific expressions of behaviour (exploring, feeding, resting and hiding) over a 24 hour period. We compared behaviour among woodlice marked with nail polish and queen-bee marker with the unmarked control group during a nine-day experiment. Although we did not find any influence of marking on survival, there was an evident influence on behaviour in most cases. Generally, in the groups of marked individuals of *Armadillidium vulgare* there were large differences observed against the control group in the overall activity. Activity of marked individuals was significantly reduced and they preferred hiding. The influence of polish and marker on the overall frequencies of behavioural categories was evident, mainly in feeding, resting and hiding. The influence on the frequency of exploring was significant in the polish marked group only.

## Introduction

From time to time zoologists need to distinguish individuals of model species. Individual identification is important in ecological studies (e.g. migration or population size) as well as in ethological studies (e.g. home range or social hierarchy). Researchers are able to use individual phenotypic/genotypic differences to identify individuals of some vertebrate species (cf McGregor and Peake 1998) but this approach is a waste of time in studies of animals with short life spans such as many invertebrates. Several methods of marking invertebrate animals have been developed. Internal marking methods used in invertebrates are based generally on colouring and are suitable mainly for unpigmented animals (e.g. termites, tiny spiders or woodlice). Other internal marking methods are based on using isotopes (radioactive or stable ones) but they are limited mainly to population studies (Southwood and Henderson 2000). Paris (1965) also used this method in a study of common pill woodlouse dispersal. More frequently external marking methods are used in studies of invertebrates. They are especially used for marking of adult insects. Beside scarification (e.g. deformations of beetle elythrae by rasper or laser) and tagging (labels with code on locusts, molluscs etc.), painting is one of the most popular methods of external marking. Painting of woodlice has been used during laboratory and field studies of their life history (Lawlor 1976, Madhavan and Shribs 1981), shelter fidelity (Brereton 1957, den Boer 1961) and vagility (Paris and Pitelka 1962). A typical substance used for marking woodlice has been “enamel”, substituted by nail polish in the study of Madhavan and Shribs (1981).

Acceptable methods for animal marking should not affect survival (such as increasing probability of predation or infection, or causing intoxication) or behaviour of marked individuals. The potential influence of marking on survival of marked animals is often evaluated but the influence on behaviour is generally neglected (cf Gallepp and Hasler 1975). Hence we decided to investigate if external marking could influence the behaviour of the common pill woodlouse, *Armadillidium vulgare* (Latreille, 1804) using two external marking methods: nail polish and queen-bee marker. Our study also aimed to investigate the potential influence of marking on survival.

## Materials and methods

### Biological material and marking process

Common pill woodlice, *Armadillidium vulgare*, were hand-collected in Olomouc City (Czech Republic). Collected animals of similar size were sorted out and reared in plastic boxes under room conditions (approx. temperature 21°C, almost 100% air humidity in boxes, natural summer photoperiod, sufficient raw potato food, stones as shelters). Three groups of 40 individuals were chosen for the experiment. Both first and second group were marked, the third group was left unmarked and served as a control.

The two external markings selected for the experiment were nail polish (60 seconds RIMMEL London^TM^) and queen-bee marker (Uni Paint Marker^TM^). The fast-drying nail polish was selected to reduce the probability of bonding tergites or sticking of an individual to the surface. Animals were picked up gently with two fingers, marked quickly with a small dot of marking agent on the first pereion segment and placed back into the box. The control group was also manipulated (i.e. picked up and placed in a box, but without marking agent).

### Experimental design

The experiment was performed during August 2009. Individuals from polish-marked, marker-marked and control groups were placed in groups of 4 to a box (box size 20×20×10 cm with 0.5 cm layer of plaster of Paris). A box with 4 randomly chosen individuals from one group was considered as one sample. Each box was divided into thirds: the first third contained 3 shelters made from dark but see-through red plastic, the second third contained 40 g of fine soil and the last third contained 3 pieces of potatoes as food. After sunset a red coated flashlight was used to minimize the disturbance of individuals. There were 10 repetitions of each treatment, i.e. 30 boxes altogether. After the marking process, individuals were left to acclimatize in the experimental boxes for 2 days. Observations were performed for 24 hours on the 3^rd^, 6^th^ and 9^th^ day after marking. The actual behaviour of each individual was recorded once each hour with the naked eye. Active behavioural categories were recorded as: *exploring* (walking), *monitoring* (staying with moving antennae), *cleaning* (clearing of antennae or legs), *interacting* (contact with another individual outside soil or shelter) or *feeding* (feeding on potato, excrements or soil, drinking or defecation). Inactive behavioural categories were recorded as *hiding* (inactivity in soil or in shelter) or *resting* (inactivity on surface).

### Statistical analysis

The effect of marking on survival of woodlice was tested by comparing the number of dead individuals from groups using a Fisher’s Exact Test. To study behavioural responses to treatment, each behavioural category was defined as proportion of individuals from the group of 4 individuals in the same box exhibiting this particular type of behaviour. The four commonest (see below) categories of behaviour were evaluated, i.e. *feeding*, *exploring*, *resting* and *hiding*. Because time of day clearly acts as a strong confounding variable with a non-linear effect on behaviour of animals during the day, we decided to include this variable in the model structure. We analysed the effect of treatment (3 levels: control, marker and polish) on proportions of the exhibited type of behaviour by fitting generalized additive models (GAMs) which are capable of accounting for nonlinearity imposed by time of day, thereby leaving residuals for category testing. We set binomial error distribution and logit link function to model the effect of both predictors. We used package *mgcv* in program R (Wood 2006) which is exceptional by solving the smoothing parameter estimation problem as part of the estimation procedure. This procedure also provides approximate p-values for the null hypotheses that each term is zero. We modelled behavioural activities for the 3^rd^, 6^th^ and 9^th^ day separately. The smoothing term for time of day was always significant justifying the presence of this variable in the model. The effect of marking on *activity* (we analysed the main active categories, feeding and exploring, jointly) was visualized in program Oriana for Windows and also analysed with GAMs.

## Results

We did not find any difference between survival of woodlice from the control group when compared with woodlice from the polish-marked group (3 *vs* 1 dead individual in these groups; *p*=0.615) or with woodlice from the marker-marked group (3 *vs* 0 dead individuals; *p*=0.241).

In total, 8640 records of behaviour were collected, but some behaviour categories were recorded rarely (*cleaning* 25 times, *interacting* 37 times, *monitoring* 88 times). Influence of marking on behaviour was evident in most cases at first sight: animals looked apathetic (i.e. they moved slowly and were less disturbed during manipulations than the controls).

**Figure 1.**
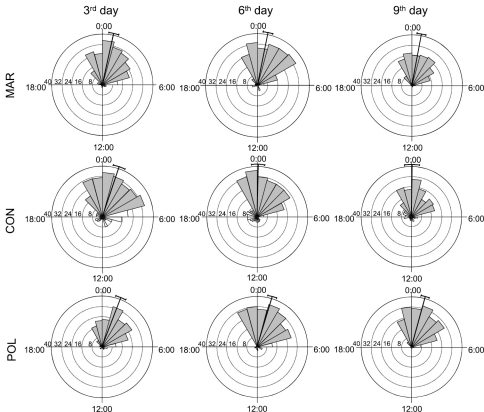
Time-distribution of active behavioural categories (feeding and/or exploring) of *Armadillidium vulgare* from all groups in observational days. Legend: CON – control, MAR – marker-marked, POL – polish-marked, grey triangles mark night-time activity, black line running from the centre of the diagram to the outer edge marks mean time of activity and the arcs extending to either side represent the 95% confidence limits.

There are differences evident between *activity* of woodlice from control group and woodlice from both marked groups (Fig. 1). Woodlice were active mainly during night, although a few unmarked individuals were active during the daylight as well. Their activity generally started between 21:00 and 22:00 and finished at 05:00. Peaks of activity were between 00:00 and 01:30 (Fig. 1). Activity of woodlice from both marked groups was significantly lower in all observation days (with the exception of polish-marked group in the last day, Table 1) and showed the same daily pattern.

**Table 1.** Statistical tests for each level of treatment that the estimate differs from zero. Whereas parameter estimate for control group was estimated as intercept, parameters for level marker and polish represent pure effects. Significance testing was carried out after accounting for variation imposed by time of day. Behavioural category activity represents joined evaluation of both active categories (i.e. feeding and exploring) (see Fig. 1).

		activity		resting		feeding		exploring		hiding	
		*z value*	*p*	*z value*	*p*	*z value*	*p*	*z value*	*p*	*z value*	*p*
3rd day	*control* (intercept)	-9.30	< 0.001	-17.87	< 0.001	-17.17	< 0.001	-19.33	< 0.001	-4.68	< 0.001
	*marker* (x *control*)	-6.43	< 0.001	-8.52	< 0.001	-4.51	< 0.001	-1.45	0.147	12.34	< 0.001
	*polish* (x *control*)	-8.91	< 0.001	-5.22	< 0.001	-6.09	< 0.001	-2.64	0.008	11.93	< 0.001
6th day	*control* (intercept)	-11.93	< 0.001	-19.50	< 0.001	-16.58	< 0.001	-15.69	< 0.001	0.77	0.444
	*marker* (x *control*)	-4.48	< 0.001	-2.61	0.009	-3.18	0.001	0.09	0.932	6.00	< 0.001
	*polish* (x *control*)	-3.34	< 0.001	-5.33	< 0.001	-5.30	< 0.001	3.75	< 0.001	7.18	< 0.001
9th day	*control* (intercept)	-15.06	< 0.001	-19.15	< 0.001	-16.88	< 0.001	-19.06	< 0.001	7.59	< 0.001
	*marker* (x *control*)	-4.49	< 0.001	-4.83	< 0.001	-4.19	< 0.001	-1.02	0.306	7.39	< 0.001
	*polish* (x *control*)	-1.12	0.264	-4.67	< 0.001	-4.42	< 0.001	3.22	0.001	4.45	< 0.001

**Figure 2.**
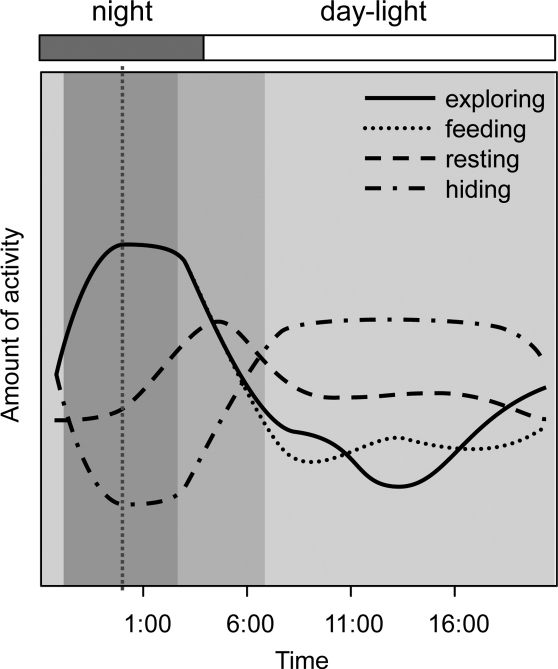
Daily patterns of behavioural categories as modelled by fitting GAM to illustrate a high degree of non-linearity in the response (logits). Compound graph from curves expressing frequency of exploring, feeding, resting and hiding of *Armadillidium vulgare* in a mean day

All main behavioural categories were recorded with a significant 24 hour pattern. The typical daily patterns of behavioural categories of *Armadillidium vulgare* were visualized without effect of marking and effect of experimental day using GAMs (Fig. 2).

**Figure 3.**
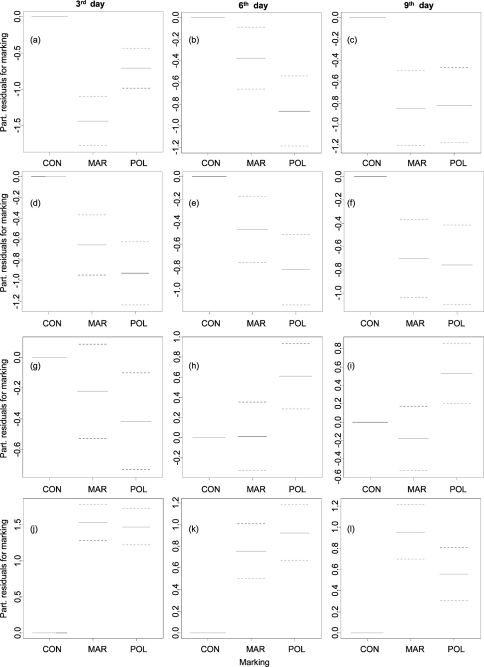
Influence of marking on frequency of *resting* (a), (b), (c), on *feeding* (d), (e), (f), on *exploring* (g), (h), (i), and on *hiding* (j), (k), (l) of *Armadillidium vulgare* in 3^rd^, 6^th^ and 9^th^ day analyzed by GAMs (confidence intervals dotted). Legend: CON – control, MAR – marker-marked, POL – polish-marked.

*Resting* of woodlice was recorded mainly before sunrise (c. 05:00–06:00, Fig. 2). Woodlice from both marked groups rested significantly less during the whole experiment (Figs 3a–c, Tab. 1). Resting was the least frequent behaviour category among evaluated ones; woodlice were recorded resting 846 times. *Feeding* was generally the second most frequented category (1023 recorded acts) of behaviour, woodlice fed regularly at c. 00:00–05:00 (Fig. 2). Nevertheless feeding was significantly decreased by marking; individuals from both marked groups fed less in contrast to unmarked ones in all three days (Figs 3d–f, Tab. 1). *Exploring* behaviour of woodlice (recorded 982 times) showed a typical and significant daily pattern in spite of marking; woodlice were exploring boxes during night and feeding at the same time (Fig. 2). Although there were no significant differences in the frequency of exploring between woodlice from marker-marked group and woodlice from control group, woodlice marked by nail polish exhibited significantly less exploring in the 3^rd^ day and more exploring in following days (Figs 3g–i, Table 1). *Hiding* was the most frequent behaviour (5523 recorded acts). Woodlice were hidden especially during daylight (c. 06:00–21:00, Fig. 2). Marked woodlice were hidden in shelters significantly and strikingly more frequently compared with unmarked woodlice (Figs 3j–l, Table 1).

## Discussion

We evaluated the effect of two external marking agents (nail polish and queen-bee marker) on behaviour and survival of the common pill woodlouse *Armadillidium vulgare*. Neither agent had any effect on survival of woodlice, but influence on behaviour was evident in almost all studied cases. Woodlice of both marked groups were less active, with less feeding and more hiding in contrast to those from the control group. Woodlice marked by nail polish also exhibited less exploring at 3^rd^ day.

Den Boer (1961) used marking of woodlice (*Porcellio scaber* Latreille, 1804) by “shellac-solution in alcohol with pigment” to study shelter fidelity. He marked woodlice found on trees and tried to observe them again an hour later. He saw only about 10–20% of them (even though he prevented them escaping from the trees using tree-banding grease) and he concluded that the marked woodlice were hidden in shelters on tree trunks. Similarly our marked woodlice from both groups exhibited more hiding over the whole experiment. Their hiding behaviour could be connected with aggregation as result of attraction between conspecifics (Devigne et al. 2011) as well as looking for excrement as suitable source of food (Hassall and Rushton 1982). Greater exploring behaviour of unmarked woodlice at start of experiment can be associated with active interest in the new neighbourhood, marked animals were more apathetic.

Paris and Pitelka (1962) using marked woodlice (*Armadillidium vulgare*) found that the population is very fluid. They observed only a few marked individuals in bait traps the day after marking. At first sight, this is contrary to our results. Nevertheless from the activity pattern of *Armadillidium vulgare* it is evident that they are hiding during daylight and feeding/exploring during night. Paris and Pitelka checked their traps during nights, i.e. during feeding/exploring. Probably the marked animals were hidden somewhere else and did not enter trap due to lower activity and lower level of feeding.

Common pill woodlice were significantly less active due to marking. Cuticle of terrestrial isopods is relatively permeable to water, they avoid desiccation by finding a locality with suitable humidity, e.g. shelter during daytime (Hornung 2011). In our parallel study with the pill millipede, *Glomeris tetrasticha* Brandt, 1833, marked individuals were also significantly less active than unmarked ones. Moreover, this effect of marking on activity was much more intensive compared with the results presented here about *Armadillidium vulgare* (Drahokoupilová and Tuf 2011). Perhaps we could search for the reasons in anatomy. The thin cuticle of *Glomeris tetrasticha* is very permeable for water (Edney 1951) in comparison with thicker cuticle of *Armadillidium vulgare*. We suppose some chemicals from polish and marker might break through cuticle into haemolymph of pill woodlice as well as pill millipedes. Lower activity and higher resting could have been a result of some poisoning overshoot. This question should be explored. The queen-bee marker probably did not affect behaviour of marked bees, because the dot of marking agent is not in contact with cuticle but usually only with hairs (Sammataro and Avitabile 1978). The lack of evidence for effect of marking on survival of woodlice should be interpreted carefully. Firstly, we evaluated effect of marking on survival and behaviour for 9 days only. We do not know if marked woodlice will show higher mortality later or not. Late increased mortality could be caused e.g. by reduced feeding activity of marked woodlice. Secondly, we found an effect of external marks of nail polish on survival of woodlouse *Porcellio scaber* in a longer experiment recently (Tuf et al., in prep.).

Our observations about night activity of *Armadillidium vulgare* are supported by previous studies. Refinetti (2000) found that *Armadillidium vulgare* shows strongly nocturnal activity under a natural light-dark cycle, more or less controlled by an endogenous timer (Cloudsley-Thompson 1956, Smith and Larimer 1979).

We conclude that common pill woodlice should not be externally marked by nail polish or by queen-bee marker. Both marking agents cause lower activity of marked woodlice and their usage, for example in capture-mark-recapture studies, can provide biased or wrong results.
